# Giant laryngeal granuloma developed after a severely stressful life event

**DOI:** 10.1002/ccr3.3387

**Published:** 2020-09-29

**Authors:** Jacopo Ettori, Alessandra D'Onghia, Lorenzo Pignataro, Giovanna Cantarella

**Affiliations:** ^1^ Otolaryngology Department Fondazione IRCCS Ca' Granda Ospedale Maggiore Policlinico Milan Italy; ^2^ Department of Clinical Sciences and Community Health Università` degli Studi di Milano Milan Italy

**Keywords:** airway obstruction, dysphonia, giant laryngeal granuloma, globus, psychological stress

## Abstract

Laryngeal granulomas are ascribed to laryngopharyngeal reflux, voice abuse, and endotracheal intubation, but their pathogenesis is controversial. A recurrent giant granuloma causing dyspnea occurred after a severe psychological stress and was successfully treated by surgery, steroid injection, and psychotherapy. This case highlights the role of psychological stress in granulomas pathogenesis.

## Graphical abstract

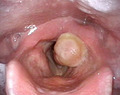

Laryngeal granulomas are commonly ascribed to laryngopharyngeal reflux, voice abuse, and/or endotracheal intubation trauma, but their pathogenesis is still controversial.^1^

A 21‐year‐old man presented at our laryngology clinic with a history of three previous microlaryngoscopic surgeries performed elsewhere for a huge laryngeal granuloma, with short‐term recurrences despite medical treatment (oral steroids and proton‐pump inhibitors) and voice rehabilitation. His symptoms occurred at the age of eighteen after a severe psychological stress, due to the devastating hemorrhagic stroke of his 5‐year‐old brother.

He complained of persistent dysphonia, effort dyspnea, globus sensation, and chronic throat clearing of respiratory obstruction with progressive worsening. Videolaryngoscopy demonstrated a bulky granuloma arising from the medial surface of the left arytenoid and ventricular band (Figure 1), significantly obstructing the supraglottis.

**FIGURE 1:**
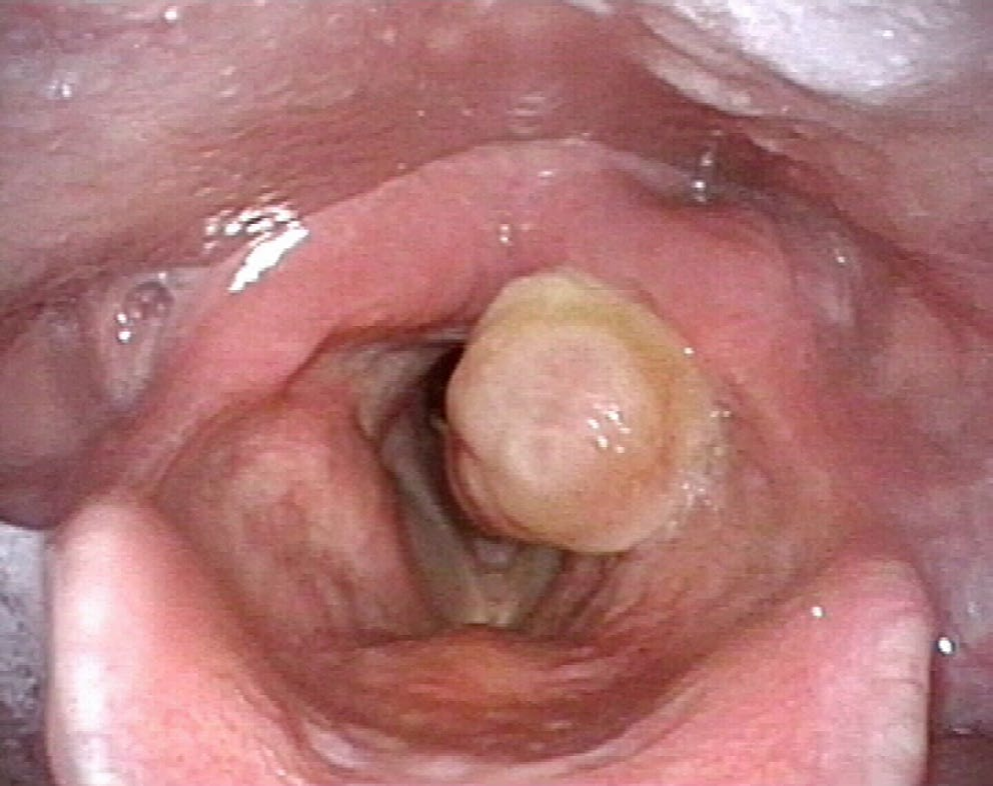
Videolaryngoscopy demonstrating a giant left laryngeal granuloma

The mass was removed under direct microlaryngoscopy by cold instruments and electrocoagulation of its vascular pedicle (Figure 2); triamcinolone acetonide was injected into its base. Histopathology showed squamous mucosa with underlying granulation tissue and acute inflammation, consistent with laryngeal pyogenic granuloma. After surgery, psychotherapy and further voice rehabilitation were carried out; no recurrence was detected at 12‐month follow‐up (Figure 3).

**FIGURE 2:**
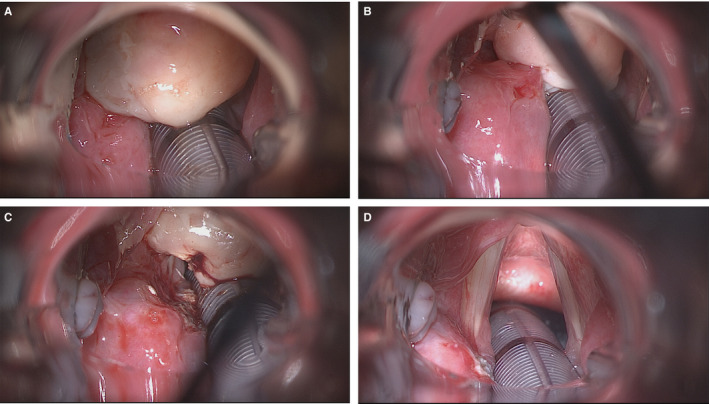
A, Microlaryngoscopic view showing the granulomatous mass which significantly obstructs the airway lumen. B, The granuloma is medially retracted, showing its base of implant on the left arytenoid and ventricular band. C, After monopolar cauterization of its vascular pedicle, the granuloma has been detached D, End of surgery, showing patency of the laryngeal inlet

**FIGURE 3:**
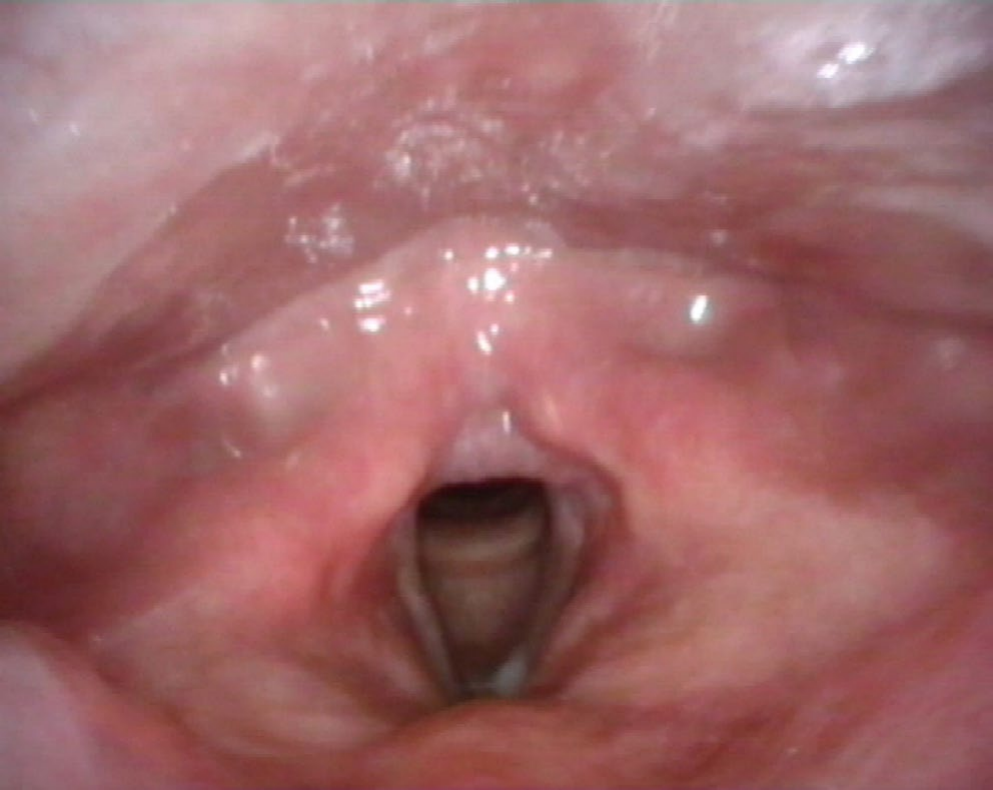
Videolaryngoscopic view 6  mo after surgery: normal laryngeal morphology and motility were observed

The described case highlights the possible role of stressful life events in the pathogenesis of idiopathic laryngeal granulomas.^2^

## CONFLICT OF INTEREST

None declared.

## AUTHOR CONTRIBUTIONS

JE**:** contributed to design of the work, manuscript drafting, participation in surgery, and patient follow‐up. ADO: contributed to manuscript revision and participation in surgery. LP: contributed to manuscript revision and clinical supervision; GC: contributed to design of the work, revision of the manuscript, performing surgery, and patient follow‐up.

## ETHICAL APPROVAL

Approval by the Ethical Committee of the IRCCS Fondazione Ca' Granda Ospedale Policlinico di Milano was obtained.
